# BMP9 is a potent inducer of chondrogenesis, volumetric expansion and collagen type II accumulation in bovine auricular cartilage chondroprogenitors

**DOI:** 10.1371/journal.pone.0294761

**Published:** 2023-11-22

**Authors:** Oliver F. W. Gardner, Yadan Zhang, Ilyas M. Khan

**Affiliations:** 1 Stem Cells & Regenerative Medicine, Great Ormond Street Institute of Child Health, University College London, England, United Kingdom; 2 Faculty of Medicine, Health & Life Science, Swansea University Medical School, Wales, United Kingdom; Università degli Studi della Campania, ITALY

## Abstract

Reconstruction of the outer ear currently requires harvesting of cartilage from the posterior of the auricle or ribs leading to pain and donor site morbidity. An alternative source for auricular reconstruction is in vitro tissue engineered cartilage using stem/progenitor cells. Several candidate cell-types have been studied with tissue-specific auricular cartilage progenitor cells (AuCPC) of particular interest. Whilst chondrogenic differentiation of competent stem cells using growth factor TGFβ1 produces cartilage this tissue is frequently fibrocartilaginous and lacks the morphological features of hyaline cartilage. Recent work has shown that growth factor BMP9 is a potent chondrogenic and morphogenetic factor for articular cartilage progenitor cells, and we hypothesised that this property extends to cartilage-derived progenitors from other tissues. In this study we show monoclonal populations of AuCPCs from immature and mature bovine cartilage cultured with BMP9 produced cartilage pellets have 3-5-fold greater surface area in sections than those grown with TGFβ1. Increased volumetric growth using BMP9 was due to greater sGAG deposition in immature pellets and significantly greater collagen accumulation in both immature and mature progenitor pellets. Polarised light microscopy and immunohistochemical analyses revealed that the organisation of collagen fibrils within pellets is an important factor in the growth of pellets. Additionally, chondrocytes in BMP9 stimulated cell pellets had larger lacunae and were more evenly dispersed throughout the extracellular matrix. Interestingly, BMP9 tended to normalise the response of immature AuCPC monoclonal cell lines to differentiation cues whereas cells exhibited more variation under TGFβ1. In conclusion, BMP9 appears to be a potent inducer of chondrogenesis and volumetric growth for AuCPCs a property that can be exploited for tissue engineering strategies for reconstructive surgery though with the caveat of negligible elastin production following 21-day treatment with either growth factor.

## Introduction

The complex morphological structure of the outer ear means that it is a particularly difficult to reconstruct after trauma, tumour resection or because of congenital deformity [1, 2]. The degree of reconstruction that is required can vary greatly and depends on the cause and size of the defect as well as the tissues that are affected. Smaller defects requiring the replacement of cartilage can be reconstructed using composite grafts of skin and auricular cartilage from the posterior conchal bowl [1]. However, to reconstruct larger defects, or to reconstruct a whole outer ear (in the case of microtia/anotia) costal cartilage is used [1, 2]. Costal cartilage is typically harvested from the seventh to nineth rib, as the natural shape of this autograft helps in producing an accurate framework when implanted subcutaneously during reconstructive auricular surgery [2–4]. Six months after the initial surgical procedure a second is performed to elevate the framework from the side of the head [2, 4]. Both of these reconstructive techniques require autologous cartilage to effect the repair, and, costal cartilage grafting in particular can lead to pain and deformity at the donor site as well as carrying the risk of pneumothorax during harvesting [3]. Alternatives to donor cartilage have been sought in the form of alloplastic implants, but despite some success a suitable candidate material has not been found [5, 6]. As a result of this there is a particular need for an alternative source of cartilage tissue that can be used for the reconstruction of auricular defects [3].

A potential source of auricular implants for surgical reconstruction is in vitro tissue engineered cartilage made from stem or progenitor cells. A range of cell types have been investigated for cartilage tissue engineering including bone marrow derived mesenchymal stem cells [7], adipose derived stem cells [8, 9] and chondrocytes, which are currently used for autologous chondrocyte implantation for articular cartilage repair [10, 11]. Due to the difficultly associated with replicating the elastic nature of auricular cartilage, particularly when using non-native cells, auricular chondrocytes (ACs) and auricular chondroprogenitor cells (AuCPCs) are of particular interest for tissue engineering approaches for the reconstruction of the outer ear [12–15].

The presence of cartilage-specific chondroprogenitors in postnatal articular tissues was first predicted following experiments using the marsupial *Monodelphis domesticus* whose foetuses grow ex utero. Bromodeoxyuridine pulse-chase labelling of developing foetal articular cartilage revealed the presence of a slow cycling subpopulation of chondrocytes in the surface zone [16]. Earlier studies using human foetal cartilage found surface zone chondrocytes express significantly higher levels of the fibronectin receptor integrin α5β1 [17, 18], this then allowed progenitors to be enriched as colony forming units from enzymatically isolated chondrocytes from immature articular cartilage using differential adhesion to fibronectin [18, 19].

Chondroprogenitor cells (CPC) fulfil the general criteria used to identify mesenchymal stromal cells (MSC), including plastic adherence, surface marker expression, adipogenic, osteogenic and chondrogenic differentiation plasticity [20, 21]. CPCs are less susceptible to senescence during in vitro expansion and can undergo chondrogenic differentiation after more than thirty population doublings [22, 23]. Subsequently, auricular chondroprogenitor cells (AuCPCs) have been isolated from equine, murine, porcine and, most recently, from human auricular cartilage [3, 14, 15, 24, 25]. AuCPCs similarly display the characteristics of mesenchymal stem cells and can generate cartilage tissue in vitro [14, 25]. AuCPCs for auricular reconstruction can be harvested from a small section of cartilage from the posterior of the conchal bowl for small defects [1] or from the cartilage rudiment in the case of patients with microtia. Microtic auricular cartilage contains AuCPCs and their capacity to form histologically normal cartilage is currently being investigated [25], although there is debate whether AuCPCs from microtic rudiments can produce cartilage tissue matching that produced by chondrocytes from normal auricular cartilage [26–28].

Transforming growth factor-β (TGFβ) was first shown to stimulate cartilage production in MSCs by Seyedin, Thomas [29]. Johnstone, Hering [7] refined the chondrogenic medium formulation by supplementing it with ascorbic acid, dexamethasone and insulin-transferrin-selenium. TGFβ-induced chondrogenesis has become the standard method to assess the cartilage forming capacity of receptive cells [30]. However, studies have shown that TGF growth factors may not be optimal inducers of chondrogenesis in MSCs and that bone morphogenetic factor-9 (BMP9) is a more potent differentiation factor for articular cartilage-derived CPCs than either TGFβ1, -2 or -3 [31]. Morgan, Bauza-Mayol [31] showed that differentiation using bone morphogenetic factor-9 (BMP9) results in greater matrix deposition producing larger pellets and increased organisation that better reflects the structure of native cartilage. Additionally, BMP9 has differential effects on chondrocytes depending on the developmental stage of tissues [32].

In light of these findings, we hypothesised that BMP9 is capable of inducing chondrogenesis and volumetric expansion in other cartilage-derived progenitors and in this study, we chose auricular-derived chondroprogenitors to test this claim using TGFβ1 as a comparator. By isolating AuCPCs from immature and mature tissue we were also able to determine if they elicited differential responses to growth factor stimulation. We compared the effect of BMP9 and TGFβ1 on AuCPCs using histological, immunological, biochemical and gene expression analyses.

## Materials and methods

Isolation and culture of auricular chondroprogenitors: Auricular chondroprogenitors were isolated from tissue collected from the central pinnae of seven day old calves, referred to as immature AuCPCs, and eighteen to twenty-four month old cows, referred to as mature AuCPCs (Cig Calon Cymru, Wales, UK). Swansea University is registered for use of animal by-products as required under the requirements of Article 23 (EU) No, 1069/2009, and the work carried out in this study using these products was following institutional approval. The pinnae were first deskinned and the perichondrium removed before being minced and sequentially digested using 0.2% pronase (Roche) in Dulbecco’s modified Eagle’s medium (DMEM, Gibco, UK) for one hour at 37°C followed by 0.06% collagenase from Clostridium histolyticum (Sigma) in DMEM with 10 mM HEPES pH 7.5 (ThermoFisher, UK), 50 μg ml^-1^ gentamycin (ThermoFisher, UK), 1% foetal bovine serum (FBS, Invitrogen, UK) for sixteen hours at 37°C. AuCPCs were selected from the total chondrocyte population via differential adhesion to fibronectin as described by Dowthwaite, Bishop [18]. Briefly, after digestion the full depth chondrocyte population was passed through a 40 μm cell strainer (Corning, UK) to produce a single cell suspension, the cells were counted and one thousand cells were then transferred into each well of a fibronectin coated six well plate (Greiner, UK, 100 μg ml^-1^ fibronectin in phosphate buffered saline with 1 mM CaCl_2_ and 1 mM MgCl_2_ left overnight at 4°C to coat then washed in warmed DMEM, Sigma, UK) for twenty minutes. The cell suspension was then removed, and expansion growth medium added (DMEM low glucose with sodium pyruvate and glutamate (Gibco, UK) 10% FBS, 50 μg ml^-1^ gentamicin, 10 mM HEPES pH 7.5 and 50 μg ml^-1^ ascorbate-2-phosphate (Sigma, UK)). After seven days colonies containing more than thirty-two cells that had formed from individual colony forming units (CFUs) were isolated with sterile cloning rings and trypsin/EDTA digestion transferred to six well culture plates containing expansion medium and expanded as clonal isolates. Clonal cell populations were cultured in growth medium for four passages to ensure sufficient cell numbers for experimental use. Clones were isolated from three separate immature (clones one to three) and mature animals (clones four to six).

Pellet culture: Pellet cultures were formed from low passage culture-expanded cells of individual colonies to characterise their chondrogenic response to TGFβ1 and BMP9. Briefly, cells were trypsinised and counted before being resuspended at 500,000 cells ml^-1^ in differentiation medium. One millilitre of cell suspension was then transferred to 1.5 ml Eppendorf tubes and centrifuged at 375 x g for 10 minutes. Pellets were left to round-up into a sphere for three days before the medium was changed. Pellets were cultured for 21 days at 37°C and 5% CO_2_ and the medium was changed twice a week. Control pellets were cultured in a differentiation medium consisting of DMEM (high glucose with sodium pyruvate and glutamate), 10% FBS, gentamicin, 50 μg ml^-1^ L-proline and 100 μg ml^-1^ ascorbate-2-phosphate. TGFβ1 and BMP9 stimulated groups were cultured in differentiation medium supplemented with 10 ng ml^-1^ TGFβ1 or 100 ng ml^-1^ BMP9 (both PeproTech EC Ltd, UK). Conditioned culture medium was collected from pellets at each medium change and was pooled by week for analysis. Twelve pellets were produced from one immature and one mature donor to provide five pellets for biochemical analysis, four pellets for gene expression and three pellets for histology. A further two immature and mature clones, all from different animals, were used to generate pellets for histological analysis. Biochemical and gene expression analysis were therefore performed on technical replicates from one clone, whilst histology and surface area measurements were performed on biological replicates from three immature and three mature clones.

Sample processing: Auricular cartilage tissue collected during cell isolation and pellets for histology were fixed in 10% NBF (Sigma, UK) for 24 hours and then transferred to PBS before paraffin wax embedding. Pellets for biochemical analysis were washed in PBS then digested in 300 μg ml^-1^ papain (from Papaya latex, Sigma, UK) in papain digestion buffer (20 mM sodium acetate pH 6.8 (Sigma, UK), 1 mM ethylenediaminetetraacetic acid (EDTA), 2 mM dithiothreitol for one hour at 37°C and stored at -20°C. Pellets for gene expression analysis were digested in control medium containing 3 mg ml^-1^ collagenase (Sigma, UK) and 1 mg ml^-1^ hyaluronidase (Sigma, UK) for three hours at 37°C. The resulting cell suspensions were then centrifuged at 500 x g and the cell pellet resuspended in RLT buffer before storage at -80°C.

Histology: Formalin fixed paraffin embedded samples were sectioned to a thickness of 5 μm on a microtome prior to histological staining and immunohistochemical labelling. Sections were deparaffinised in HistoClear (National Diagnostics, USA) and dehydrated through an ethanol gradient (100% x2, 95% and 70%) before being brought to distilled water before staining. Staining for sGAG was performed with 0.1% (w/v) Toluidine Blue in water for two minutes before rinsing in several changes of distilled water and drying at 37°C for two hours. For Picrosirius red staining, sections were digested using hyaluronidase from bovine testes (1 μg ml^-1^, Sigma, UK) for one hour at 37°C. Following digestion sections were rinsed in distilled water and then stained with 0.1% (w/v) picrosirius red in saturated picric acid for two hours. For Verhoeff’s elastic stain slides were stained for fifteen minutes in stain solution consisting of 40 ml of 5% (w/v) haematoxylin in absolute ethanol, 16 ml of 10% (w/v) ferric chloride in distilled water and 16 ml of 4% (w/v) potassium iodide and 2% (w/v) iodine in distilled water. Following staining slides were briefly washed in tap water and then differentiated in 2% (w/v) ferric chloride until elastic fibres were clear under a microscope. Following each stain slides were dehydrated through an ethanol gradient, cleared in Histo-Clear, mounted using DPX (Sigma, UK) and coverslipped.

Immunohistochemistry: Sections were dewaxed in Histo-Clear and rehydrated through an ethanol gradient before being brought to deionised water. Peroxidase inactivation was then performed using 3% hydrogen peroxide (Sigma, UK) in tap water for fifteen minutes. Antigen retrieval was performed in two stages. The first stage consisted of an overnight incubation at 65°C in TRIS-EDTA buffer solution (10 mM Trizma base pH 9.0 (Sigma, UK), 1 mM EDTA disodium salt dihydrate (Sigma, UK) and 0.05% Tween 20), followed by a 1 hour treatment with 1 μg ml^-1^ hyaluronidase from bovine testes (Sigma, UK) at 37°C. Sections were blocked using RTU Normal Horse Serum (2.5%) for thirty minutes (Vector Laboratories, USA). Labelling was performed against aggrecan (1-C-6, obtained from the Developmental Studies Hybridoma Bank (DSHB), created by the NICHD of the NIH and maintained at the University of Iowa, USA), collagen type I (C2456, Sigma, UK) and collagen type II (II6B3, DSHB, USA). Primary antibodies were diluted 1:10 for aggrecan and collagen type II and 1:2000 for collagen type I, in PBS Tween-20 (0.05%). Primary antibody detection was performed using the RTU Biotinylated Pan-specific Antibody (Vector Laboratories, USA), RTU Streptavadin/Peroxidase Complex (Vector Laboratories, USA) and ImmPACT NovaRED Peroxidase (HRP) Substrate (Vector Laboratories, USA), according to manufacturer’s instructions. Sections were counterstained with Mayer’s haematoxylin (TCS Biosciences, UK), dehydrated through an ethanol gradient, cleared in HistoClear and mounted using DPX mounting medium under coverslips (Electron Microscopy Sciences, UK).

Imaging and image analyses: Images were taken using a digital eyepiece camera, Yuanj 5.0m C-mount Digital USB Microscope Eyepiece Ocular Camera attached to an Olympus BX53 light microscope. Polarised light microscopy was conducted using a Zeiss Axioscope. The quantification of the surface area of pellets was performed on images of toluidine blue stained sections using FIJI Image processing software [33].

Biochemical analyses: The 1,9-dimethyl-methylene blue (DMMB) assay was used to quantify sulphated glycosaminoglycan (sGAG) content in papain digested samples and culture medium. DMMB solution was prepared by dissolving 32 mg of 1,9-DMMB (Sigma, UK) in 20 ml of ethanol (Sigma, UK). The DMMB solution was then added to 1.914 ml distilled water, 59 ml of 1 M sodium hydroxide and 7 ml of 98% formic acid and stirred for two hours before use. A standard curve, 0–40 μg ml^-1^, was made using shark chondroitin-4-sulphate dissolved in papain digestion buffer. Papain digested samples were diluted as appropriate (in papain digestion buffer), medium samples required no dilution. Absorbance was read using a FLUOstar Omega plate reader at 525 nm. DNA quantification was performed using the QuantiT™ PicoGreen™ dsDNA Assay Kit (Thermo Fisher Scientific, UK) according to the manufacturer’s instructions. Samples were compared to a standard curve prepared using calf thymus DNA diluted to 0, 312.5, 625, 1250 and 2,500 ng ml^-1^ in papain digestion buffer. Measurements were performed at an excitation wavelength of 485 nm and emission wavelength of 520 nm using a FLUOstar Omega plate reader (BMG Labtech, UK). Total collagen content of papain digested samples was determined using the hydroxyproline assay as described by Cissell, Link [34]. 200 μl of papain digested samples were mixed with 200 μl of 4M NaOH and hydrolysed at 110°C overnight. Samples were then allowed to cool before being neutralised with 200 μl of 4 M HCL. Standards were prepared from hydroxyproline in papain digestion buffer at the following concentrations: 0, 6.25, 12.5, 25, 50 and 100 μg ml^-1^. Neutralised samples and standards were transferred to a 96 well micro assay plate and incubated with diluent (1:2 distilled water and propan-2-ol (Sigma, UK)) and oxidant (0.7 g chloramine T (Sigma, UK), 10 ml distilled water and 50 ml stock buffer (28.5g sodium acetate trihydrate, (Sigma, UK) 18.75 g sodium citrate dehydrate (Sigma, UK), 2.75 g citric acid (Sigma, UK), 200 ml propan-2-ol and 300 ml distilled water) on an orbital shaker for fifteen minutes. Following incubation colour reagent (7.5 g diethylamino benzaldehyde (Sigma, UK), 11.25 g 60% perchloric acid (Sigma, UK) and 62.5 ml propan-2-ol) was added and the plate incubated at 70°C for twenty minutes. The plate was then allowed to cool, and absorbance measured at 540 nm using a FLUOstar Omega plate reader.

Reverse transcription-qPCR: RNA isolation from pellets was performed using the RNaeasy mini kit (Qiagen, UK), including on-column DNA digestion using RNAse-Free DNase (Qiagen, UK) following the manufacturer’s instructions. The RNA quantity and purity within each sample was then determined using a NanoDrop spectrophotometer (ThermoFisher, UK). Complementary DNA synthesis was performed using the M-MLV reverse transcriptase (Promega, UK) with 150 ng of sample RNA as a template. The reverse transcription was then performed using a Bio-Rad CFX96 thermocycler with the following program: 25°C for ten minutes, 48°C for one hour, 95°C for five minutes and then held at 4°C. Samples were stored at -20°C until use. QPCR was performed using a Bio-Rad CFX96 thermal cycler using 20 μl reaction volumes in 96 well plates (Bio-Rad, UK). Each reaction contained 3.5 mM MgCl_2_,

200 μM dNTPs, 0.3 μM forward and reverse primers, 0.025 U μl^-1^ Taq polymerase and SYBR Green (GoTaq qPCR Master mix; A6001, Promega, UK) with programmed reaction conditions of one cycle of 95°C-10 mins followed by forty cycles of 95°C-30 s, 55°C-30 s, 72°C- 30 s and melt curve analysis.

Absolute values for gene expression were calculated from standard curves generated using cloned and sequence-verified, serially diluted, plasmid template DNA (10-fold dilutions of 1 ng to 1 fg of template per reaction). Values shown are for the gene of interest (in ng) divided by the housekeeping gene 18SrRNA (in ng). Primer sequences used for RT-qPCR have been previously published [35].

Statistical analyses: Statistical testing was performed using GraphPad Prism v.7.04. Normality testing showed that pellet area (n = 3), pellet sGAG (n = 5), pellet sGAG/DNA (n = 5) and RT-qPCR data (n = 4) was all normally distributed, as a result these data were analysed using one-way ANOVA and Tukey’s multiple comparison test. Pellet DNA (n = 5) data were not normally distributed and were analysed using Kruskal-Wallis test and Dunn’s multiple comparison test.

## Results

**Fig 1:**
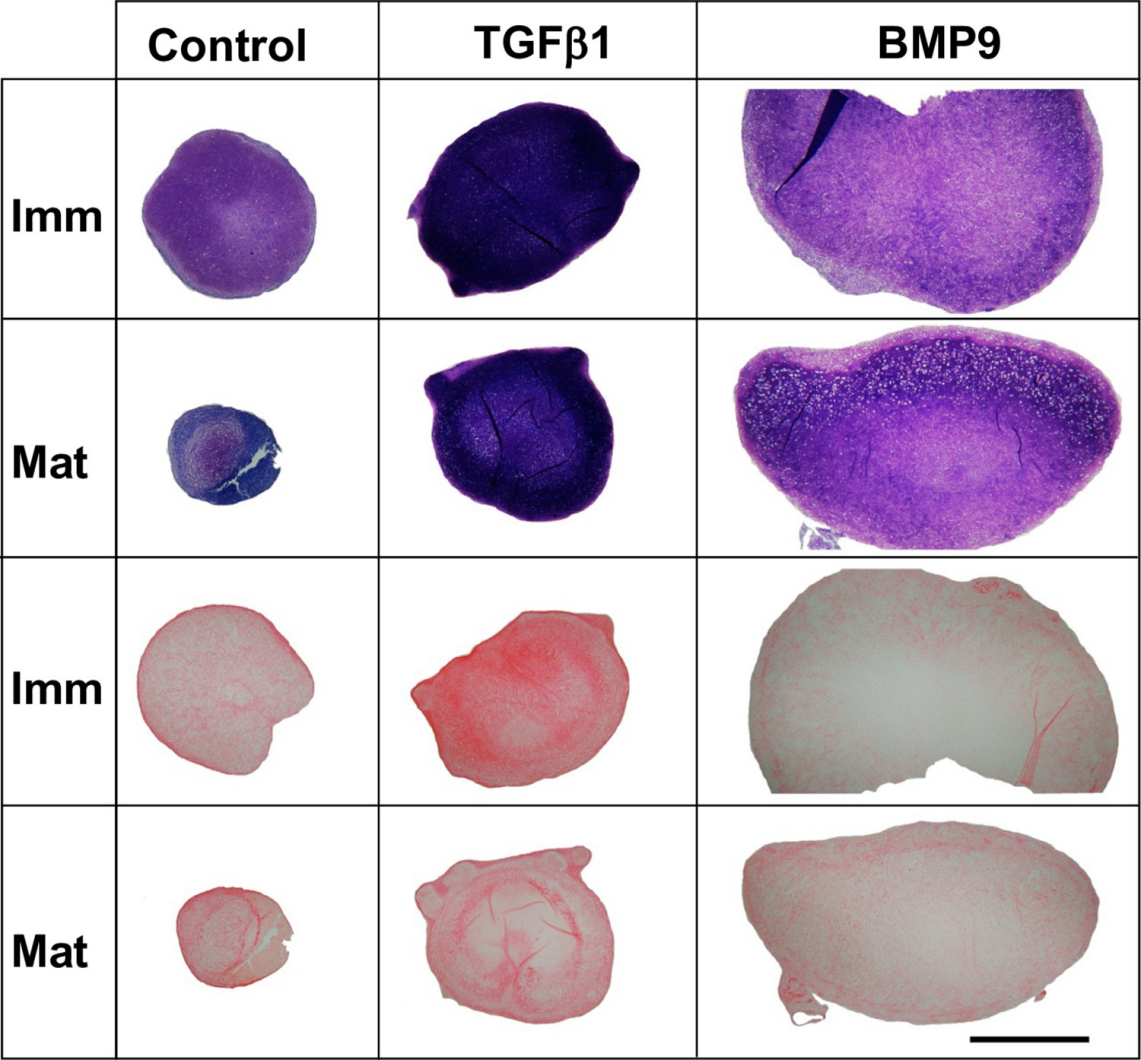
Brightfield microscopy of toluidine blue and picrosirius red staining of auricular chondroprogenitor cells cultured in high-density pellets for 21 days. Pellets from three immature and three mature clones (each isolated from different animals) containing 5x10^5^ AuCPCs were cultured in control medium or control medium supplemented with 10ng ml^-1^ TGFβ1 or 100ng ml^-1^ BMP9 to compare their chondrogenic effect. Metachromatic toluidine blue staining labels the presence of sGAG in cultured cell pellets. Picrosirius red staining shows localisation and organisation of collagen fibrils. Scale bar represents 1000 μm.

Clonal chondroprogenitor cell isolates from immature and mature bovine pinnae (n = 3) were differentiated in chondrogenic medium for 21 days and processed for histology (Fig 1). During culture BMP9 pellets grew noticeably larger than TGFβ1 treated pellets and those in unsupplemented control medium. BMP9-supplemented cell pellets did not completely round up at the start of culture as normally occurs but adopted a more bowl-like appearance (Fig 1). TGFβ1 treated pellets were intermediate in size and consistently rounder compared to other conditions. Immature and mature-derived AuCPC cell pellets cultured in control medium exhibited a range of appearances, from small spherical balls to flaked pieces of tissue (artefacts of the disintegration of the fragile pellets during sample processing and sectioning).

Positive toluidine blue staining, indicating the presence of sGAG was found in all pellets (Fig 1). Toluidine blue staining in TGFβ1 stimulated pellets was intense with regional variation in matrix distribution where the highest intensity of staining found as a thick band around the periphery of pellets (Fig 1). Staining for sGAG in BMP9 stimulated pellets was relatively less intense and more diffusely distributed throughout the depth of pellets. Differentiated immature and mature chondroprogenitors in BMP9 stimulated pellets possessed large lacunae, especially pronounced in mature cell pellets, where chondrocytes in large chondrons surrounded by abundant extracellular matrix were located at the superior aspect of pellets. Chondrocytes in BMP9 treated pellets were evenly distributed throughout the matrix, in comparison, differentiated chondroprogenitors in TGFβ1 stimulated pellets were densely packed and cells with larger lacunae located in the centre of pellets (Fig 1). Measurement of the surface area of sectioned pellets stained with toluidine blue (Fig 1 and Fig 5A) showed that both immature and mature AuCPCs treated with BMP9 produced significantly larger pellets than controls (4.87 and 6.80fold, p≤0.0007), or those treated with TGFβ1 (3.26 and 4.95 fold, p≤0.0014). The surface area of pellets that were treated with TGFβ1 were not significantly different from those grown in unsupplemented control medium.

**Fig 2:**
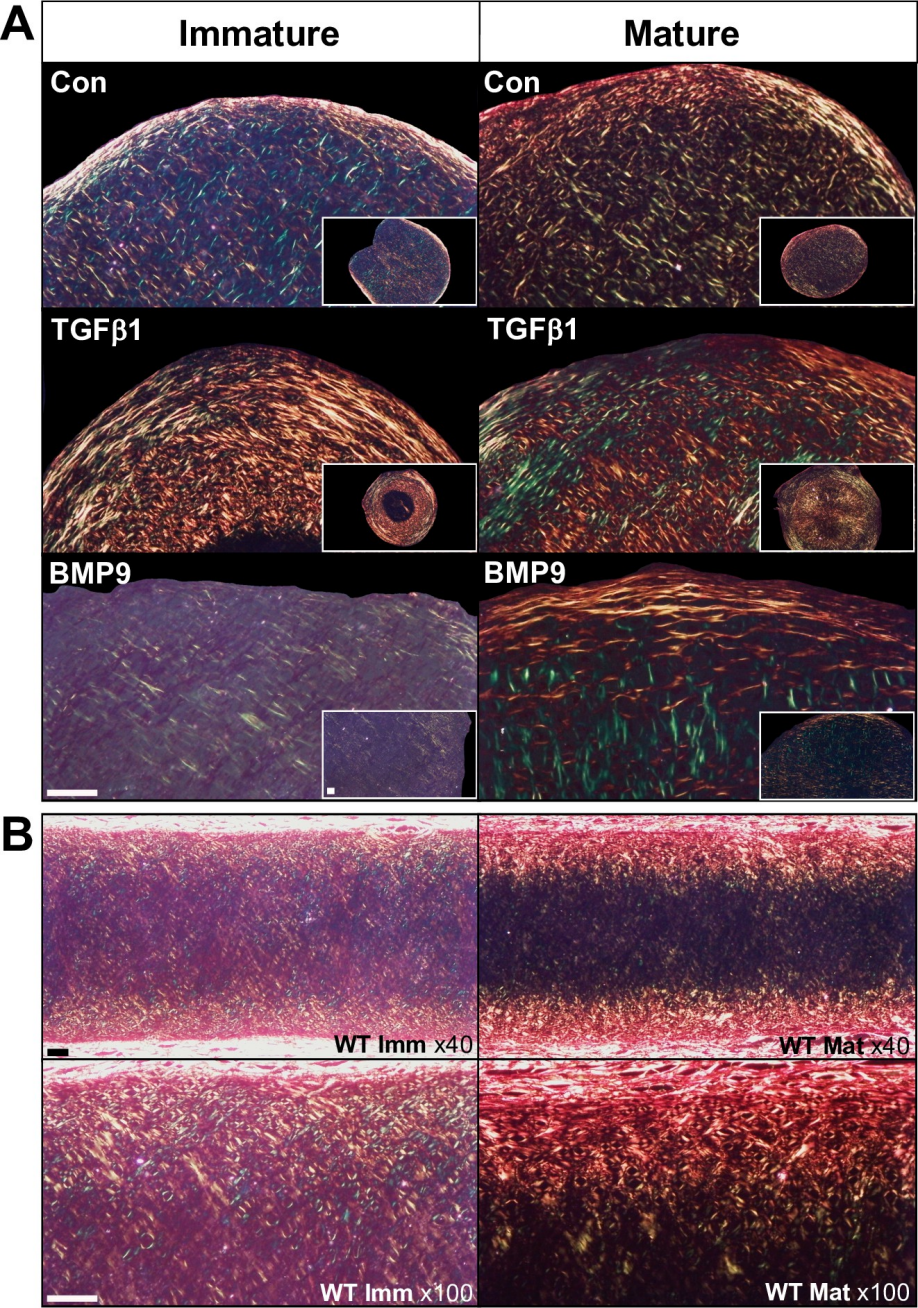
Polarised light microscopy of picrosirius red staining of auricular chondroprogenitor cells cultured in high-density pellets for 21 days and native bovine auricular cartilages. A. Representative images shown from pellets from three immature and three mature clones (each isolated from different animals) containing 5x10^5^ AuCPCs were cultured in control medium or control medium supplemented with 10ng ml^-1^ TGFβ1 or 100ng ml^-1^ BMP9. Polarised imaging of picrosirius red stained slides showed increased fibril organisation and the formation of concentric rings of fibrils in response to stimulation with TGFβ1, whist BMP9 treatment leads to more anisotropic matrix that is more representative of native tissue. B. Polarised light microscopy of native immature and mature bovine auricular cartilage showing changing collagen fibril organisation and density with age. Scale bars represent 100 μm.

Brightfield imaging of sections stained with picrosirius red (PSR) to visualise collagen showed all pellets stained for the dye (Fig 1). The distribution of collagen in TGFβ1 stimulated pellets and control pellets that underwent spontaneous differentiation was annular, with a clear outer layer rich in collagen and concentric rings of collagen towards the centre of pellets (Fig 1). PSR staining in BMP9 treated pellets was more diffuse and pericellularly located (Fig 1). Polarised light microscopy of picrosirius red stained sections of pellets (Fig 2) showed pellets cultured in control medium displayed a criss-cross arrangement of fibres in the body of the pellet with a thin band of fibres circling the periphery. In TGFβ1 treated immature cell pellets there was intense fluorescent signal from a thick peripheral band of collagen fibres with bright less organised pericellular signal penetrating deeper into the pellets. TGFβ1 treated mature cell pellets displayed the criss-cross pattern of collagen organisation, but at lower magnification a peripheral band of collagen with a deeper more radially aligned fibres were visible (Fig 2 inset). In contrast, the fluorescent signal in BMP9 treated pellets was diffuse and weaker, however in mature pellets there was evidence of green coloured fibres angled perpendicular to the surface (Fig 2). PSR staining of native immature and mature auricular cartilages revealed intense fluorescence at the peripheries of the tissue with fibres predominately aligned in parallel orientation with the intensity progressively decreasing towards the centre of the cartilage where fibres were more pericellularly located (Fig 2B). The colour of fluorescent fibres, green, red or yellow, does not reflect different collagen types or other fibrous proteins, colour changes denote changes in collagen organisation or orientation [36].

**Fig 3:**
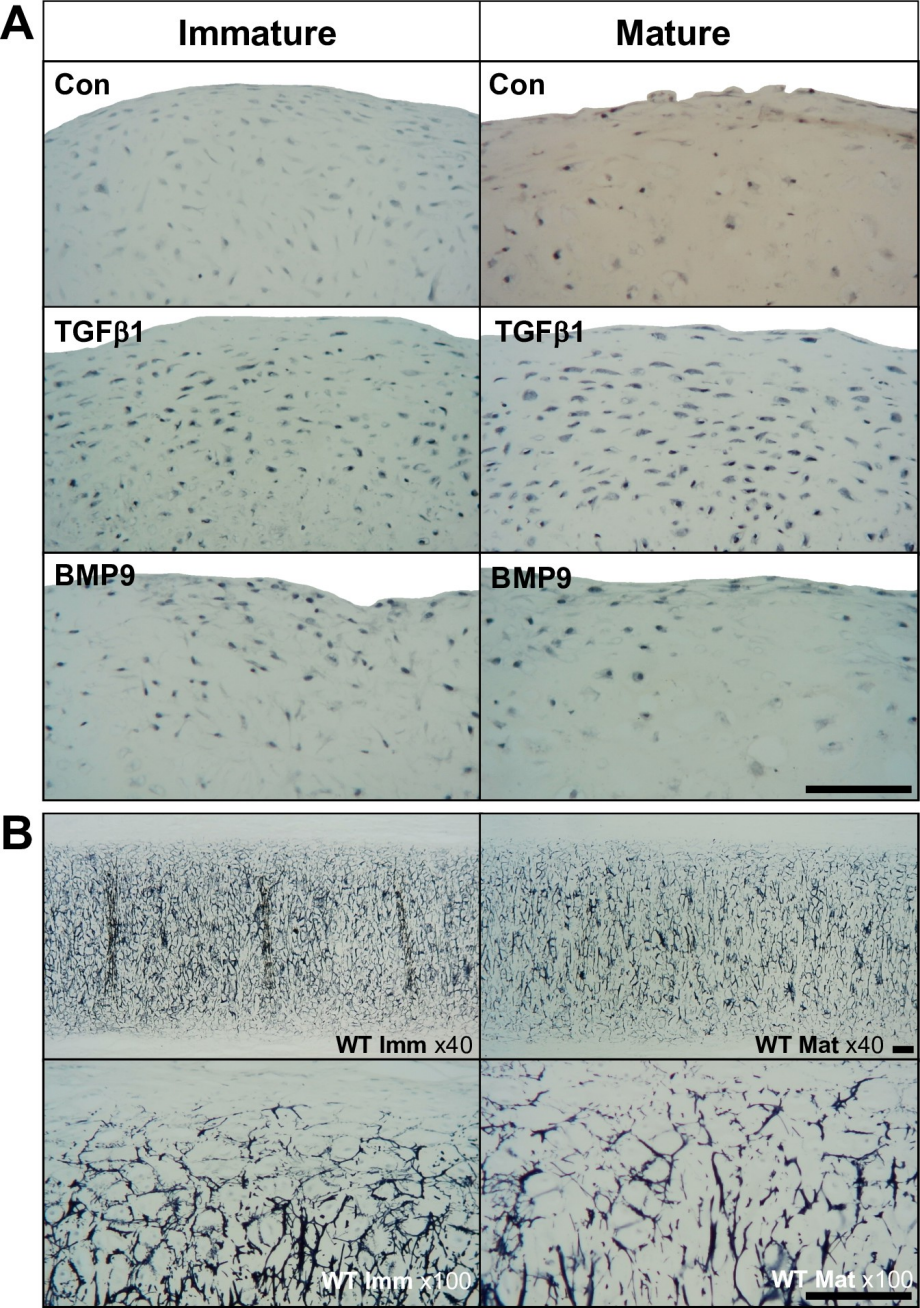
Verhoeff elastic cartilage staining of auricular chondroprogenitor cells cultured in high density pellets for 21 days and native bovine auricular cartilages. A. Representative images shown for pellets from three immature and three mature clones containing 5x10^5^ AuCPCs cultured in control medium or control medium supplemented with 10ng ml^-1^ TGFβ1 or 100ng ml^-1^ BMP9. Neither treatment led to the deposition of extracellular elastin fibres, but increased intra/pericellular staining was seen in response to TGFβ1. B. Staining of native bovine auricular tissue shows a decrease in the density and interconnectivity of elastic fibre networks with age. Scale bars represent 100 μm.

Treatment with BMP9 or TGFβ1 did not lead to extracellular matrix deposition of elastic fibres as determined by histological staining in chondroprogenitor differentiated pellets compared to the level observed in native tissues (Fig 3A and 3B). However, TGFβ1 treatment led to increased intra- and pericellular Verhoeff histochemical staining compared to BMP9 treated and control pellets (Fig 3A). Staining for elastin in native immature and mature bovine auricular cartilages showed a dense extracellular pattern of elastin fibres with a slight decrease in staining evident with increasing age (Fig 3B).

**Fig 4:**
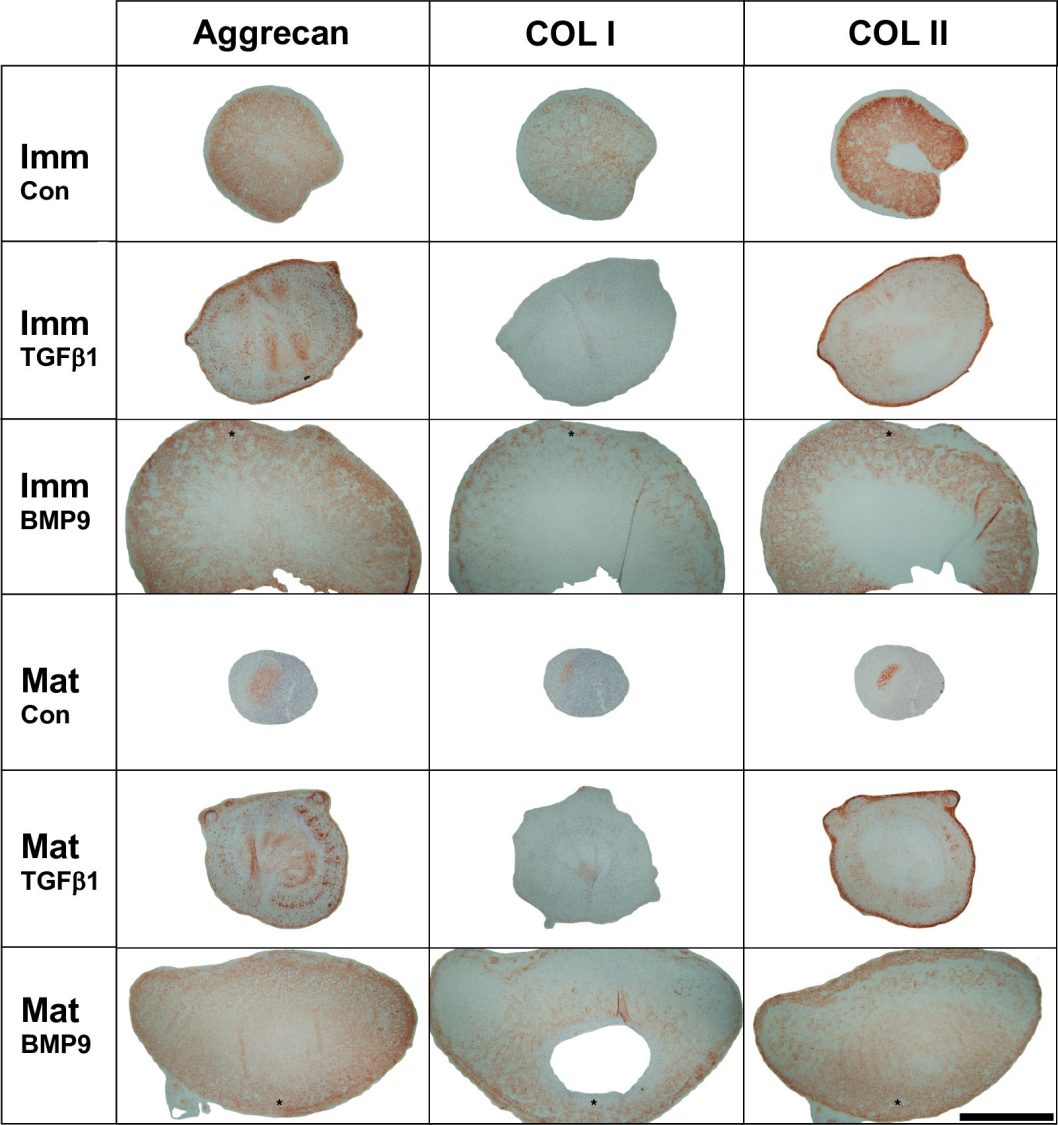
Immunohistochemical labelling of aggrecan in extracellular matrix produced by auricular chondroprogenitor cells cultured in high-density pellets for 21 days. Immunolabelling showed that culture with BMP9 and TGFβ1 and unsupplemented medium demonstrate aggrecan and collagen type II deposition. In contrast, little, if any, collagen type I was found in TGFβ1 stimulated pellets and only deposited in low levels of around the periphery of BMP9 stimulated pellets. The inferior aspect of MP9 treated pellets is shown by an asterisk. Scale bar represents 1000μm.

Immunohistochemical labelling for aggrecan in mature and immature BMP9 treated pellets antibody binding was diffusely distributed throughout the extracellular matrix with a gradual increase in labelling evident with greater depth, a pattern replicated in immature pellets cultured in control medium (Fig 4). In TGFβ1 stimulated pellets aggrecan mainly labelled the periphery of pellets or in patches in more central regions of pellets (Fig 4). Collagen type I labelling was found in control pellets that had undergone robust chondrogenesis and were intact and at low levels in BMP9 stimulated pellets, concentrated more in the periphery in their inferior aspects, whilst it was present at low levels pericellularly in TGFβ1 stimulated pellets (Fig 4). The distribution of collagen type II was starkly different between BMP9 and TGFβ1 stimulated pellets (Fig 4). In immature and mature TGFβ1 pellets collagen type II labelling was highly concentrated at the periphery of pellets, more diffuse but weak labelling was present deeper within pellets. In BMP9 stimulated immature pellets collagen type II labelling was present as a diffuse wide band extending from the periphery and midway into the pellet with the highest levels of staining in the inferior aspects of pellets (Fig 4 - *asterisks*). Collagen type II labelling was noticeably present in immature compared to mature pellets cultured in control medium.

**Fig 5:**
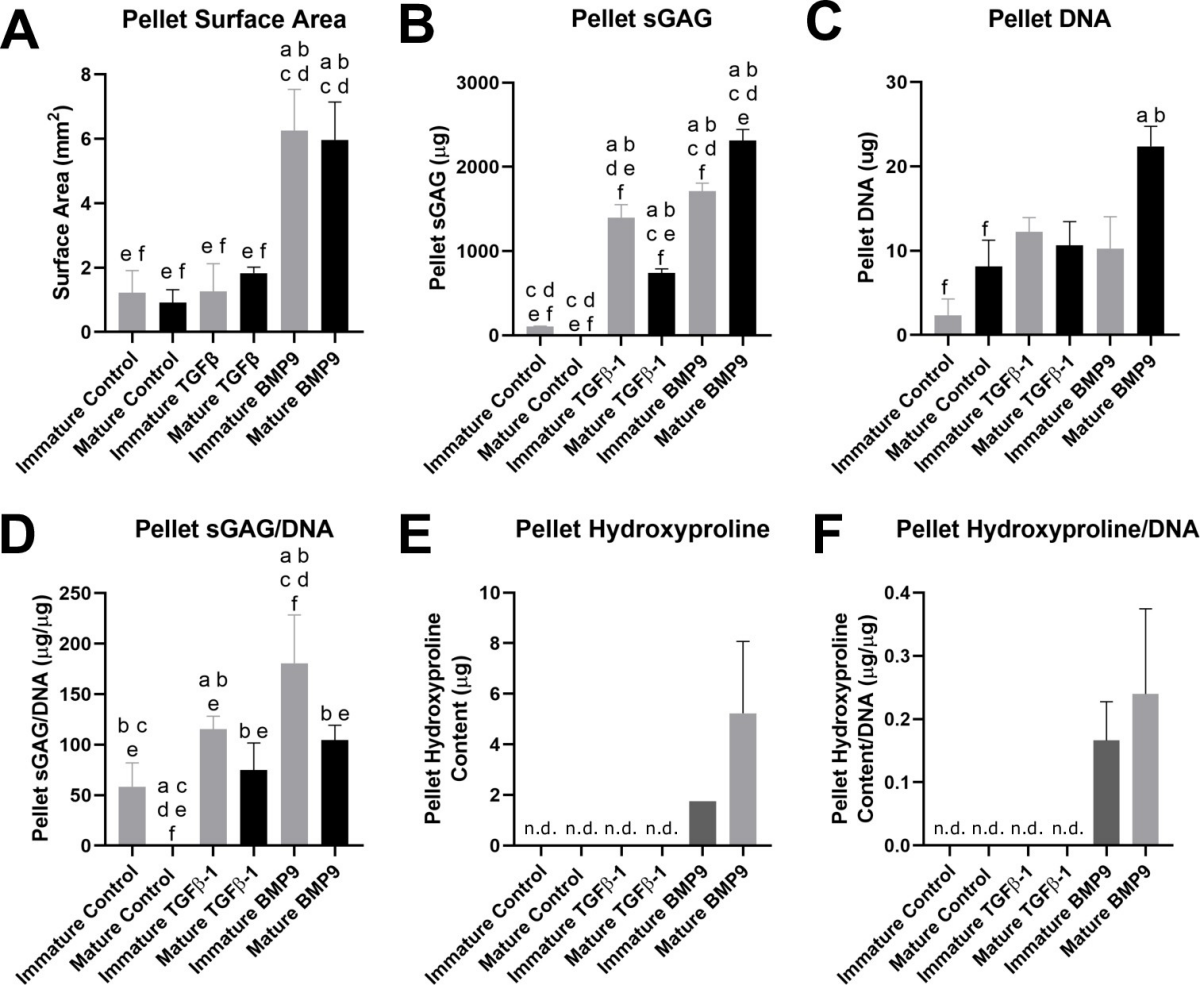
Quantitative surface area and biochemical analyses of high-density pellets of auricular chondroprogenitors after 21 days in culture. The surface area of sectioned pellets stained with toluidine blue was quantified using Image J (A; n = 3). Analysis of the sGAG deposited in each of the pellets was performed using the DMMB assay (B; n = 5), the DNA content of each pellet was quantified using the Quant-iT™ PicoGreen™ dsDNA Assay Kit (C; n = 5) and the hydroxyproline in each pellet quantified using hydroxyproline assay (E. n = 3–5). Results of sGAG and hydroxyproline are also presented as normalised to the DNA content of each pellet (D. and F.). Samples in which no hydroxyproline was detected are labelled ‘n.d.’. Significance was taken as p<0.05, ‘a’ denotes a significant difference to ‘Immature control, ‘b’ to ‘Mature control’, ‘c’ to ‘Immature TGFβ1’, ‘d’ to Mature TGFβ1’, ‘e’ to Immature BMP9’ and ‘f’ to ‘Mature BMP9’.

Biochemical analysis of the raw amounts of sGAG deposited in pellets showed that it was significantly increased in response to TGFβ1 and BMP9 compared to controls for both immature (13.63 and 16.66-fold respectively, p<0.0001, n = 5) and mature differentiated AuCPC pellets (296.94 and 928.91-fold respectively, p<0.0001) (Fig 5B). In both immature and mature pellets the amount of sGAG detected in BMP9 stimulated pellets compared to TGFβ1 pellets was higher (1.22 and 3.12-fold respectively, p≤0.0003, n = 5) (Fig 5B). The sGAG detected in mature BMP9 stimulated pellets was significantly higher than that in immature BMP9 stimulated pellets (1.35-fold, p<0.0001, n = 3), conversely, the amount of sGAG in immature TGFβ1 pellets was higher than in mature pellets stimulated with TGFβ1 (1.89-fold, p<0.0001, n = 5).

Quantification of the pellet DNA content showed that there were no significant changes in cell number in immature AuCPC pellets that were stimulated with TGFβ or BMP9 (Fig 5C). Mature pellets stimulated with BMP9 contained significantly more DNA than control pellets (2.75-fold, p = 0.038, n = 5).

The deposition of sGAG normalised to DNA in pellets increased in response to both TGFβ1 and BMP9 in both immature (1.97 and 3.08-fold respectively, p = 0.0196 and p<0.0001, n = 5) and mature cells when compared to unsupplemented control pellets (212.52 and 296.20-fold respectively, p = 0.0014 and p<0.0001, n = 5, Fig 5D). BMP9 stimulated immature pellets contained significantly more sGAG/DNA than TGFβ1 stimulated pellets (1.56-fold, p = 0.0058, n = 5), however there was no significant difference between the effect of TGFβ1 and BMP9 on mature AuCPC pellets. The amount of sGAG/DNA within the immature control (195.87-fold, p = 0.0163, n = 5) and BMP9 stimulated pellets (1.72-fold, p = 0.0011, n = 5) was significantly higher than in both the respective mature groups.

Analysis of sGAG released into the medium over three weeks of culture with TGFβ1 and BMP9 indicated a steady loss of proteoglycan into the medium by both immature and mature AuCPC cell pellets, with BMP9 having a greater effect that TGFβ1. Variations within the total sGAG (medium + pellet) normalised to DNA data were large (*data not shown*), they did however show increasing trends in total sGAG production in response to BMP9 in the immature pellets and an increase in production in response to both growth factors in mature pellets.

Hydroxyproline content normalised to DNA was consistently detectable only in immature (1.74 ± 0 μg/μg^-1^ hydroxyproline/DNA, n = 3) and mature (5.23 ± 2.83 μg/μg^-1^ hydroxyproline/DNA, n = 5) BMP9 treated pellets. Hydroxyproline levels were below the level of sensitive for our assays for all other pellets except for a single mature TGFβ1 stimulated pellet (Fig 5E and 5F). The hydroxyproline content for BMP9 treated immature and mature pellets was not significantly different.

**Fig 6:**
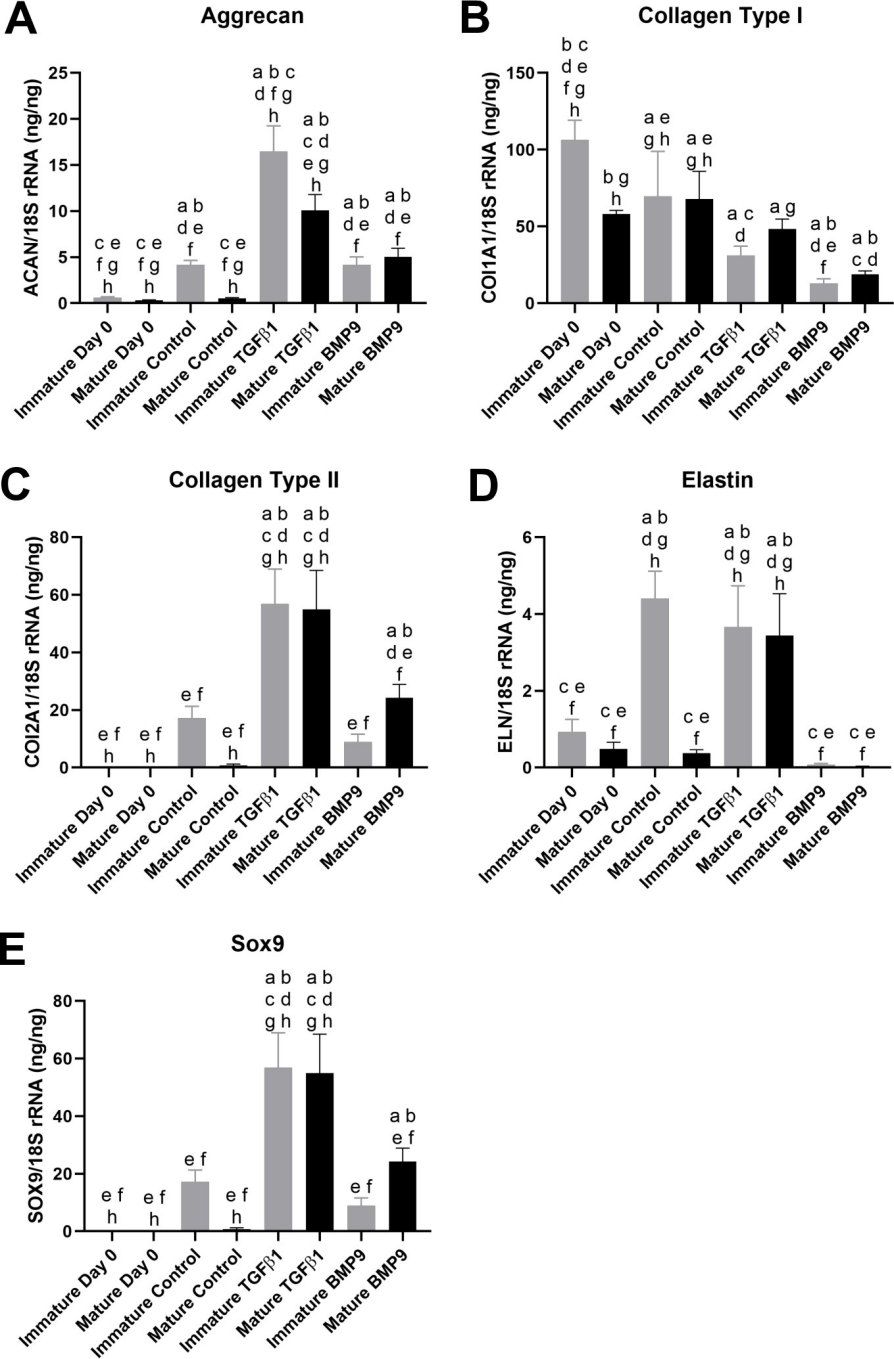
Quantitative gene expression of auricular chondroprogenitor cells cultured in highdensity pellets for 21 days. Pellets from one immature (n = 4) and one mature clone (n = 4) containing 5x10^5^ AuCPCs were cultured in control medium or control medium supplemented with 10ng ml^-1^ TGFβ1 or 100ng ml^-1^ BMP9. Cells from each donor were also collected at the point of seeding as a ‘Day 0’ time point (n = 3). Gene expression of A. ACAN, B. COL1A1, C. COL2A1, D. ELN and E. SOX9 were determined using reverse transcription quantitative real-time PCR. Significance was taken as p<0.05, ‘a’ denotes a significant difference to ‘Immature Day 0’, ‘b’ to ‘Mature Day 0’, ‘c’ to ‘Immature Control, ‘d’ to ‘Mature Control’, ‘e’ to ‘Immature TGFβ1’, ‘f’ to Mature TGFβ1’, ‘g’ to Immature BMP9’ and ‘h’ to ‘Mature BMP9’.

Reverse transcription real-time PCR analysis demonstrated clear differences in gene expression between groups sampled at the end of the 21-day culture period. Expression of the cartilage matrix components aggrecan (ACAN), collagen type II (COL2A1) and SRY-box transcription factor-9 (SOX9) was significantly higher (p≤0.0004, p≤0.001 and p≤0.001 respectively, n = 4) in TGFβ1 stimulated pellets than control and BMP9 pellets (Fig 6A–6E). No significant differences were seen between immature and mature pellets within TGFβ1 or BMP treated groups for ACAN, COL2A1 or SOX9. However, ACAN expression was significantly higher in immature compared to mature pellets cultured in control medium (8.1fold, p = 0.0132, n = 4) (Fig 6A). Elastin, a key matrix component of elastic auricular cartilage was found to be transcribed at a significantly higher level in immature control as well as both TGFβ1 treated cell types compared to all other groups (p≤0.0008) (Fig 6D.). In contrast the expression of collagen type I (COL1A1) was highest on day zero (although lower in mature cells than immature cells) and in control groups at day 21 of culture and significantly lower in TGFβ1 and BMP9 stimulated groups at this time point (p≤0.026) (Fig 6B).

## Discussion

These data show that the capacity of auricular progenitors to differentiate and undergo volumetric expansion is potently enhanced by BMP9. Of note was the significantly increased pellet size and collagen content in BMP9 treated cell pellets compared to stimulation with TGFβ1. Morgan et al demonstrated that BMP9 is more potent in inducing differentiation of articular cartilage-derived chondroprogenitors than a number of other chondrogenic factors including TGFβ1–3, and that it has context-dependent roles in cartilage development, specifically in inducing important aspects of postnatal maturation such as collagen reorientation [31]. Hills et al showed that BMP9 has differential effects on immature and mature explanted articular cartilages, stimulating matrix production in the former and having comparatively little or no effect on the latter [32]. Here we demonstrate that BMP9 is a potent inducer of chondrogenesis of both immature and mature auricular chondroprogenitors acting in a differential manner to TGFβ1, as shown the by quantity, distribution, and organisation of their synthesised extracellular matrices. Additionally, AuCPC pellets from mature cells were able to form large lacunae characteristic of adult cartilage indicating the phenotypic stability following extended culture.

The data presented in this study show BMP9 and TGFβ1 induced chondrogenic differentiation of AuCPCs is not equivalent in several significant ways. Whilst they are both members of the TGFβ superfamily, BMP9 and TGFβ1 act via different signalling pathways [37]. TGFβ1 signals through ALK5 receptors and signalling is transduced through SMAD 2/3 [38], in contrast BMP9 signals through ALK1/2 receptors and SMAD 1/5/8 [39] or non-SMAD (MAPK; p38, ERK and JNK) signalling pathways (reviewed by Herrera, Dooley [40]). The effects of these two divergent signalling pathways on auricular progenitors probably explain the different chondrogenic responses seen in this study.

The data presented in this paper allow us to make some generalisations when using tissue-specific chondroprogenitors about the effects of these growth factors on chondrogenesis, specifically tissue size. Firstly, BMP9 led to increased deposition of both sGAG (aggrecan) and collagen type II, demonstrated by the results of both biochemical and immunohistochemical analysis, with matrix production driving increased pellet size. It is useful to note that whilst immunohistological screening with anti-collagen type II antibodies does not show significant differences in the amount of deposition, biochemical analysis is more definitive. Secondly, BMP9 pellets contain chondrocytes within well-defined and large lacunae surrounded by extracellular matrix, reflecting the appearance of native auricular cartilage [25], whereas TGFβ1 stimulated pellets were characterised by densely packed cells that were difficult to resolve except at higher magnification. Hypertrophy of articular chondrocytes during development has been shown to play a role in the growth of articular cartilage [41], a similar effect is likely to contribute to the increase of pellet size caused by BMP9 stimulation. Third, the clear differences in the localisation and organisation of collagen type II and aggrecan within BMP9 and TGFβ1 treated pellets may partially explain the difference in pellet size. In TGFβ1 pellets, histological staining and immunohistochemical labelling showed a thick band of proteoglycan (sGAG) deposition and highly organised annular rings of collagen most prominent in the periphery of pellets. This typical pattern of extracellular matrix deposition has also been seen in previous studies using TGFβ1 to stimulate chondrogenesis in articular and auricular cartilage derived progenitor pellet cultures [22, 25, 31]. In contrast, the extracellular matrix deposited in BMP9 treated pellets is more diffusively distributed. In normal cartilage tissue at rest, the tensile pressure of the collagen network constrains the osmotic swelling pressure of proteoglycans. If the collagen network is labile, i.e. during growth or in pathological conditions such as osteoarthritis, collagen tensile forces are reduced, the consequences are increased hydration and volumetric growth until equilibrium is reached. It is likely therefore collagen networks of BMP9 pellets are less stiff and more hydrated than TGFβ1 pellets. Evidence for this assertion is two-fold; first, the diffuse nature of both proteoglycan and collagen localisation within extracellular matrices shown in this study, and second, previous work using atomic force microscopy showing extracellular matrices of BMP9 treated chondroprogenitors are more elastic than TGFβ1 treated cell matrices [31]. Therefore, a major difference between TGFβ1and BMP9 induced chondrogenesis of auricular progenitors we hypothesise is the tensile forces exerted by their collagen networks. TGFβ1 matrices are arranged concentrically within pellets creating a highly tensile collagen network (as shown by polarised light microscopy of PSR stained sections) that resists the osmotic swelling of highly concentrated proteoglycans. BMP9 induces a differently organised collagen type II network that allows expansion under the influence of the swelling pressures created by proteoglycans until they are balanced, allowing the formation of a larger pellet. The fluorescence signal aligning perpendicular to the BMP9 treated pellet surfaces may indicate either collagen fibril reorientation by radial volumetric growth or the pattern of fibril deposition which then influences the trajectory of growth.

Studies have shown that modulating the collagen fibril network in dynamic systems can stimulate volumetric growth. Asanbaeva, Masuda [42] experimentally reduced the tensile mechanical integrity of collagen using beta-aminopropionitrile (an inhibitor of collagen crosslinking) and induced significant volumetric growth in in vitro cultured immature cartilage explants. The same mechanism applies to osteoarthritic tissue, where regions of intact cartilage exhibit a greater capacity to swell and hydrate than normal cartilage indicating imperceptible early damage to the collagen network [43]. In normal cartilage, once growth has ceased and the opposing tensile and swelling forces are in balance, the collagen matrix undergoes maturation to fix the network in place for the lifetime of the organism [44–46] requiring the activation of a specific set of developmentally encoded proteins such as LOXL1 [35].

Another potentially interesting finding was BMP9 was able to induce chondrogenesis more consistently in immature cell lines than TGFβ1. This variable response to growth factors was not seen in mature clones stimulated with TGFβ1 in which the response was consistent, suggesting greater heterogeneity in phenotype between immature progenitor clones. There was also variation in the chondrogenic response of all cells cultured in control medium, ranging from non-formation of pellets, small non-chondrogenic pellets, to pellets that underwent chondrogenesis. This variation may be evidence of cell-intrinsic differences between progenitors and suggests that there may be multiple subtypes of progenitors within auricular cartilage–in particular immature cartilage—as has been shown for BMSC [47], chondrocytes [48, 49] and osteoarthritic articular cartilage [23].

Whilst gene expression analysis at the end of the culture period was not informative of the whole culture period, it did however highlight a major caveat when using BMP9 for chondrogenic differentiation of auricular progenitors, the lack of elastin gene expression that correlated with histochemical staining for protein. The difficulty in inducing elastin deposition during in vitro culture using culture expanded auricular chondrocytes has been documented a number of times [50–52]. The reasons for the lack of elastin expression and deposition may be linked either to incomplete differentiation of cells, or, that cells have undergone irreversible epigenetic changes upon extended (>20) population doublings solidifying their dedifferentiated phenotype [50, 53]. The fact that auricular progenitors differentiate in 3-dimensional culture producing equivalent amount of proteoglycan and significantly more collagen than TGFβ1 stimulated pellets lends more weight to the notion that chondrocytes have not fully matured. Our analysis above of BMP9 induced chondrogenesis functioning primarily to differentiate cells and increase the size of tissue also suggest only the preliminary phases of differentiation have been accomplished. Sequential addition of TGFβ1 [52, 54] following the initiation of chondrogenic differentiation by BMP9 [55] may induce maturation of the auricular chondrogenic phenotype of cells and thereby stimulate elastin deposition. The converse argument is that cells are differentiating to type, i.e. a perichondrial-like phenotype [56] where there is little or no elastin deposition in the peripheral/perichondral zone of native auricular cartilages (see Fig 3B). Given the advantages that culture expansion of tissue specific progenitors provide for tissue engineering and cell-based therapies of auricular cartilage these issues deserve to be resolved through further experiment.

For cell and tissue-based repair strategies we need robust methods for inducing chondrogenic differentiation, growth and maturation for neocartilage to be clinically useful. This study has shown that BMP9 is a potent morphological stimulator of chondrogenesis in both immature and mature auricular chondroprogenitor cells, inducing hyaline-like cartilage displaying significant volumetric expansion through homogeneous deposition of cartilage matrix proteins. These findings will help drive forward the process of designing systematic tissue engineering approaches using chondroprogenitors that are able to address the size, the maturity and functional integrity of biofabricated matrices.

## Supporting information

S1 Fig Toluidine blue staining of individual clonal cell lines from immature (clones 1–3) and mature (clones 4–6) auricular tissues grown as high-density pellets in control, TGFb1 and BMP9 supplemented culture medium. Note for immature clones BMP9 produces a consistent and robust response to chondrogenic signals when compared to TGFb1 supplemented and unsupplemented culture media.
(TIF)
