# Supplementary figures and images for: BMP9 is a potent inducer of chondrogenesis, volumetric expansion and collagen type II accumulation in bovine auricular cartilage chondroprogenitors
Source: PLoS One. 2023 Nov 22;18(11):e0294761. doi: 10.1371/journal.pone.0294761 (PMC10664884; doi:10.1371/journal.pone.0294761)

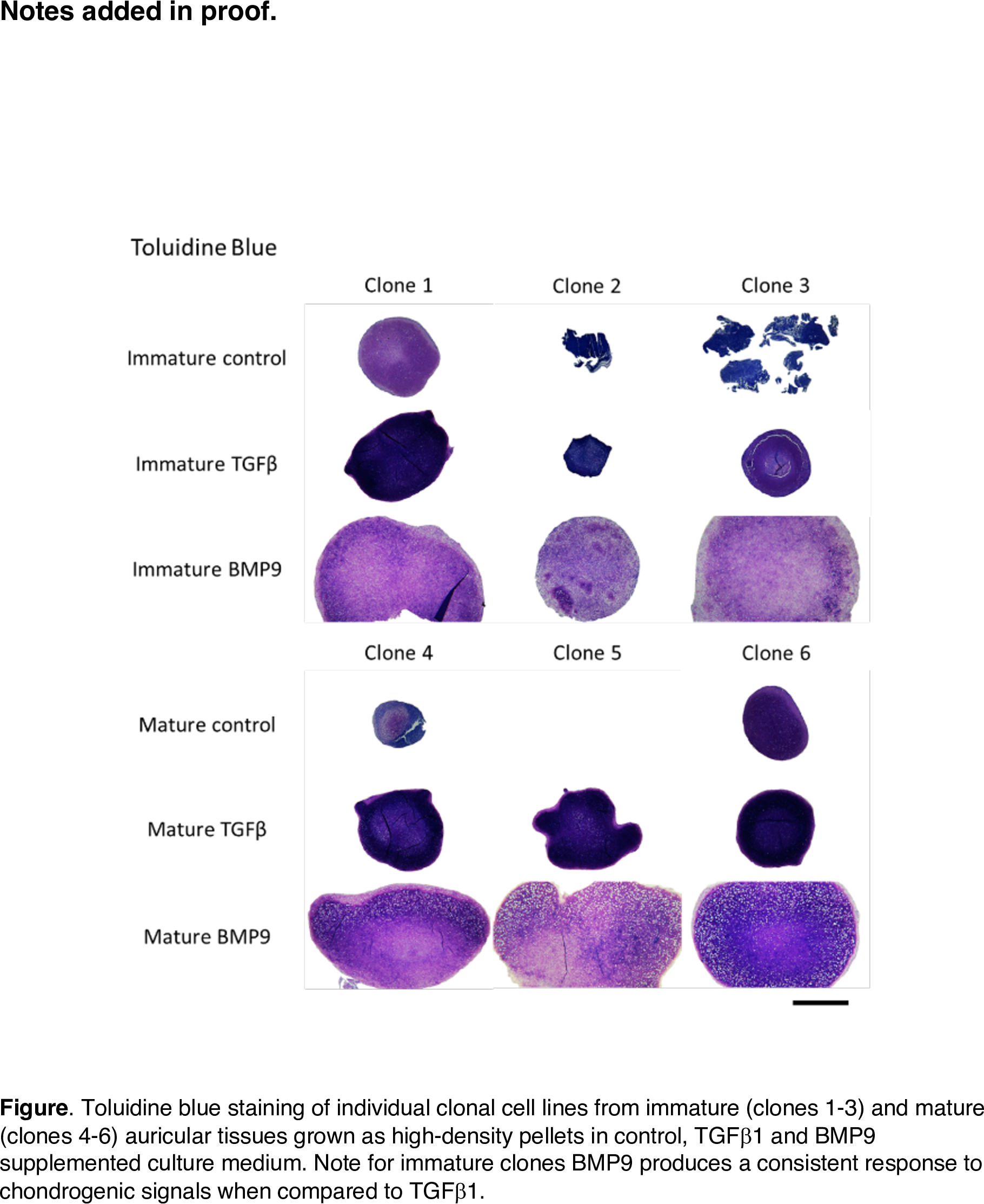

Supplement: S1 Fig — Note for immature clones BMP9 produces a consistent and robust response to chondrogenic signals when compared to TGFb1 supplemented and unsupplemented culture media. (TIF) [file pone.0294761.s001.tif]
